# Oxidative Stress and Antioxidant Treatments in Cardiovascular Diseases

**DOI:** 10.3390/antiox9121292

**Published:** 2020-12-17

**Authors:** Wenjun Wang, Peter M. Kang

**Affiliations:** 1 Beth Israel Deaconess Medical Center, Harvard Medical School, Cardiovascular Institute, Boston, MA 02215, USA; wwang4@bidmc.harvard.edu; 2 Department of Emergency, Qilu Hospital, Shandong University, Jinan 250012, China

**Keywords:** oxidative stress, reactive oxygen species, cardiovascular diseases, antioxidant defense enzymes, antioxidant molecules, microRNAs, nanoparticles

## Abstract

Oxidative stress plays a key role in many physiological and pathological conditions. The intracellular oxidative homeostasis is tightly regulated by the reactive oxygen species production and the intracellular defense mechanisms. Increased oxidative stress could alter lipid, DNA, and protein, resulting in cellular inflammation and programmed cell death. Evidences show that oxidative stress plays an important role in the progression of various cardiovascular diseases, such as atherosclerosis, heart failure, cardiac arrhythmia, and ischemia-reperfusion injury. There are a number of therapeutic options to treat oxidative stress-associated cardiovascular diseases. Well known antioxidants, such as nutritional supplements, as well as more novel antioxidants have been studied. In addition, novel therapeutic strategies using miRNA and nanomedicine are also being developed to treat various cardiovascular diseases. In this article, we provide a detailed description of oxidative stress. Then, we will introduce the relationship between oxidative stress and several cardiovascular diseases. Finally, we will focus on the clinical implications of oxidative stress in cardiovascular diseases.

## 1. Introduction

Mounting evidences show that oxidative stress has an irreplaceable role in the development and pathology of various diseases [1,2,3]. It is caused by the overproduction of reactive oxygen species (ROS), which include both the free radicals and their non-radical intermediates, such as superoxide anion (O2^•−^), hydroxyl ion (OH^•^), hydrogen peroxide (H_2_O_2_), and peroxyl radicals (ROO^•^), alkoxyl (RO^•^), singlet oxygen (^1^O_2_), and ozone (O_3_) [4]. The burst of ROS is associated with an imbalance between the generated ROS and the antioxidant defense systems. Overproduction of ROS has a detrimental role in biological system by not only targeting biological molecules, such as lipid, protein, and DNA, but also by acting as a second messenger in cellular signaling. Through targeting regulatory pathways, ROS results in cell inflammatory signals activation or programmed cell death.

Cardiovascular diseases are the leading cause of morbidity and mortality worldwide. Evidences show that oxidative stress plays an important role in the progression of various cardiovascular diseases, such as atherosclerosis, heart failure (HF), cardiac arrhythmia, and myocardial ischemia-reperfusion (I/R) injury [5,6]. A lot of work has been devoted to the studies of antioxidants therapies in prevention and treatment of these cardiovascular disease. Small molecules, such as astaxanthin and omega-3, have shown to have a beneficial role in cardiovascular diseases. While some clinical trials have shown positive results, others are controversial. The impaired function of ROS-clearance enzymes, such as superoxide dismutase (SOD), leads to high baseline levels of oxidative stress [7]. Moreover, there are new antioxidants that are being explored, and novel strategies to specifically deliver antioxidant drugs to the area of ROS overproduction [8]. In this review, we will discuss the mechanisms of oxidative stress and their therapeutic implications in cardiovascular diseases.

## 2. Methods

The literature search was performed using search terms “oxidative stress”, “cardiovascular diseases”, “antioxidants”, “myocardial I/R injury”, “HF”, “atherosclerosis”, “atrial fibrillation”, “hypertension”, “nutritional supplements”, “miRNA”, “nanoparticles”, alone or in combination. Both clinical and animal studies were included. Furthermore, publications that addressed the basic mechanisms and pathophysiology were considered. All articles included were from peer-reviewed journal in English.

## 3. Oxidative Stress

### 3.1. Generation of ROS

**Figure 1:**
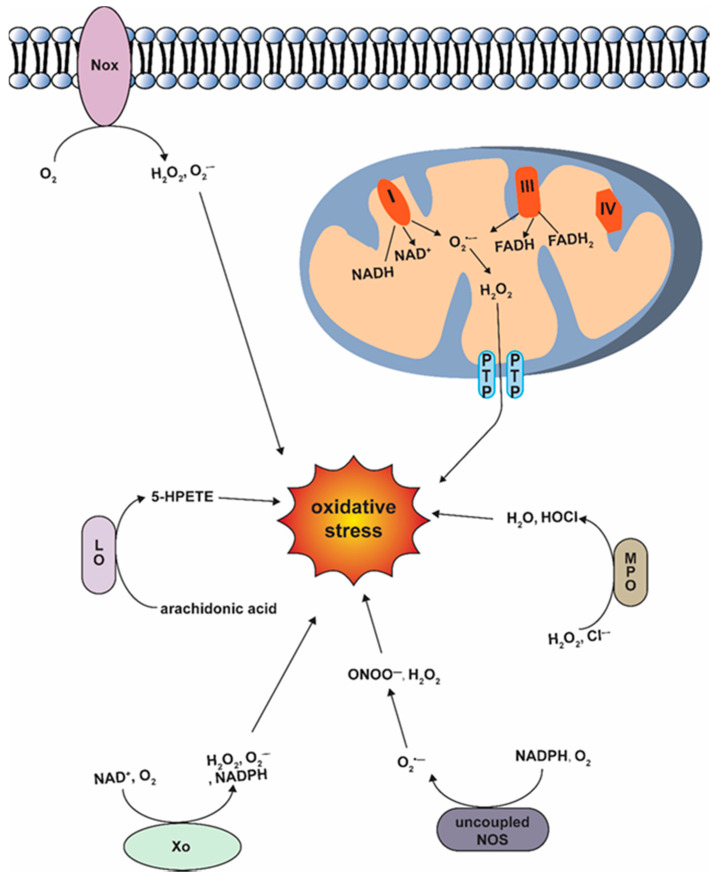
Diagrams of oxidative stress production pathways.

Mitochondria are regarded as the primary source of endogenous ROS generation through the by-products of electron transport chain (ETC) and oxidative phosphorylation [9] (Figure 1). Mitochondria, composed of the outer and the inner mitochondrial membranes and the matrix, are the major sites of adenosine triphosphate (ATP) production. Acetyl-CoA produced during tricarboxylic acid cycle is transported into the mitochondria and passed down from the complexes I, III to the complexes IV, ultimately ending up with ATP synthesis at the complex V. However, during certain pathological conditions, the mitochondrial respiratory chain is disrupted and the electron is leaked to oxygen to produce superoxide [10]. Complex I and III are regarded as the major sites of ROS production in mitochondria. There are more than 10 other enzymes that also contribute to ROS production [11]. Excessive ROS production at the mitochondria can trigger the mitochondrial permeability transition pore (mPTP) opening and disrupt mitochondrial membrane stability, which facilitates the release of ROS from the mitochondrial matrix into the cytosol [12].

Number of enzymes outside mitochondria are known to play a role in the ROS production, such as xanthine oxidase (Xo), myeloperoxidase (MPO), lipoxygenase, uncoupled nitric oxide synthase (NOS), and nicotinamide adenine dinucleotide phosphate (NADPH) oxidase (NOX) [13] (Figure 1). Among them, NOX is regarded as an important source of ROS [14]. There are more than 7 members of NOX family. NOX1, NOX2, NOX4, and NOX5 are expressed in cardiovascular system [15]. The regulation of NOX2, also known as gp91phox, is the most well-studied. The activation of NOX2 depends on the other subunits of NOX family including p22phox, p67phox, p40phox, and p47phox. The phosphorylation at Ser303, 304, and 328 leads to the activation of p47phox [16]. Then, the activated p47phox binds with p22phox, which makes p40phox and p67phox accessible to Nox2, resulting in the activation of Nox2 [17]. Once Nox2 is activated, NADPH can bind with intracellular C-terminus and transfer electrons from NADPH to oxygen to produce ROS on the other side of the membrane [18]. Additionally, Nox-derived ROS induce the activation of secondary oxidase systems including NOS uncoupling, mitochondrial dysfunction and Xo activation [19].

In addition to ROS that are produced endogenously, environmental factors could regulate exogenous ROS production. Smoking, environment pollutants, ultraviolet (UV) radiation, xenobiotics, and alcohol are the examples of exogenous sources of ROS [20]. These exogenous sources of ROS enhance ROS production through interaction with endogenous substances or enzymes. Cigarette smoke is able to activate NOX and stimulate the ROS production. UV radiation mediates the ROS production by interacting with water to produce ROS. Meanwhile, alcohol has potential to inhibit the expression of antioxidants and cytoprotective enzymes. 

### 3.2. Antioxidant Defense Enzymes

Redox homeostasis is tightly regulated by the antioxidant enzymes in the cell. Antioxidant enzymes include SOD, catalase, glutathione peroxidase (GPX), peroxiredoxin (PRX), and thioredoxin (Trx). They have an important role in defending oxidative stress by decomposing ROS [21] (Table 1). 

SOD is the only enzyme that can catalyze superoxide anion into oxygen and hydrogen peroxide [22]. Three isoforms have been identified: manganese SOD (MnSOD) located at the mitochondria matrix [23], copper-zinc SOD (Cu/ZnSOD) located at the cytoplasm and the nucleus, and extracellular SOD (ECSOD) located in the extracellular fluids [24]. Catalase can catalyze hydrogen peroxide to water. It is extensively expressed and located in peroxisomes of all types of mammalian cells except for erythrocytes and human vascular cells [25]. GPX can catalyze peroxides or organic hydroperoxides to water and oxygen, or the corresponding alcohol by glutathione [26]. Eight isoforms of GPX have been identified. GPX1 is the most ubiquitous isoform distributed in the cytosol, the nucleus and the mitochondria. GPX2 is present in the cytosol and the nucleus. GPX3 is mostly found in the cytosol. GPX4 is located at the membrane in addition to the nucleus, the cytosol, and the mitochondria [27]. 

PRX has peroxidase activity, which can catalyze superoxide peroxides, organic hydroperoxides, and peroxynitrite utilizing NADPH [37]. PRX family includes 6 isoforms containing the cysteine residues. PRX1-4 has typical 2-Cys, PRX5 has atypical 2-Cys, and PRX6 has 1-Cys [51]. Trx antioxidant family, such as Trx and thioredoxin reductase (TrxR), plays an important role in protecting cells from the oxidative stress. It is able to decrease ribonucleotide reductase and regulate the activity of redox-sensitive transcription factor resulting in DNA and protein repairing. TrxR, coupled with NADPH, could keep Trx in a reduced state [45].

### 3.3. Molecular Effects of Oxidative Stress

#### 3.3.1. Oxidative Stress and Lipids, Protein, DNA Damage

Intracellular ROS are highly active and unstable. They could cause modifications of protein, lipid, and nucleus, and modify the regulation of protein function and signal pathways. Lipids, particularly polyunsaturated fatty acids (PUFAs) and cholesterol, are considered important target substrates of oxidative stress because cell membranes are made up of lipids. In addition, the lipids are important metabolites in the cell [52]. Lipid oxidation can be divided into three steps: initiation, propagation, and termination. The initiation starts with the formation of free radicals by the unsaturated lipid molecule losing a hydrogen atom. During the propagation process, peroxyl radicals are formed by the reaction between lipid radicals and oxygen. The produced peroxyl radicals then attack the other lipids to form more lipid peroxyl radicals. When hydrogen source is all used up, a lot of non-radical products are produced [53]. Active aldehydes, such as 4-hydroxynonenal (4-HNE) and malondialdehyde (MDA), are important products of lipid peroxidation that have an important role in the pathogenesis of many diseases. The levels of active aldehydes in the blood are predictive of the disease progression [54,55].

ROS and ROS-derived lipid peroxidation are able to attack protein, a process called protein carbonylation [56]. It is caused by the combination of nucleophilic amino acids, such as cysteine, histidine, and lysine, with ROS through Michael addition process [57]. Many studies have shown that protein carbonylation is attributed to the enzyme inactivation, the degradation of proteins, and the elevated production of ROS [58].

ROS can combine with the double bonds of nucleoside bases, resulting in the formation of 8-oxo-deoxyguanosine, thymine glycol, 5-hydroxymethyluracil, 6-hydroxy-5, 6-dihydrocytosine, and 5-hydroxyuracil. Among them, 8-oxo-deoxyguanosine is the most well studied and has the potential to induce G-T transversions [59]. Furthermore, oxidative stress can regulate DNA and histone methylation to change the chromatin structure and the function of the genome [39]. Lipid peroxidation can also directly modify DNA. The damages of DNA can be repaired by nucleotide excision repair, homologous recombination, or translesion synthesis [60]. The imbalance between the damaged and the repaired DNA may lead to the transcription and translation mistakes. Evidences show that mitochondrial DNA is another important target of ROS [10]. Mitochondrial DNA is a closed-circular double-stranded DNA controlling the function of mitochondria. The attacking of mitochondrial DNA by ROS may result in the decreased mitochondrial copy and transcript numbers as well as the release of mitochondrial DNA and pro-apoptotic factors [61]. 

#### 3.3.2. Oxidative Stress and Inflammation

Inflammation is involved in the pathogenesis of many common diseases. Oxidative stress is regarded as the initiator of inflammation as well as the consequence of inflammatory responses [62]. Inflammation is also regulated by oxidative stress. Nuclear factor kappa-light-chain-enhancer of activated B cells (NF-κB) proteins are transcription factors that play a key role in the regulation of inflammation and immunity [63]. Oxidative stress can regulate the activation of NF-κB by targeting pro-inflammatory protein IκB-kinase (IKK) [64]. In addition, oxidative stress can modulate the T helper cell differentiation by interacting with T cell receptor (TCR) signaling pathways [65]. The induction of cyclooxygenase-2, NOS, and alterations in microRNAs (miRNAs) are also critical in the oxidative stress-induced inflammation [62,66]. Additionally, inflammation can enhance oxidative stress reactions. Immune cells are recruited to the damaged site resulting in “respiratory burst” and the increased release and accumulation of ROS [67]. 

#### 3.3.3. Oxidative Stress and Programmed Cell Death

Accumulative studies show that programmed cell death is a ubiquitous phenomenon in all life forms, and has a key role in various physiological and pathological processes [68]. Up to now, there are a number of well characterized forms of programmed cell death processes, such as apoptosis, autophagy, necroptosis, pyroptosis, and ferroptosis [69]. Oxidative stress plays multiple roles in regulating various kinds of programmed cell deaths. The relationship between oxidative stress and apoptosis or autophagy in the cell has been well-described [70,71]. However, the interaction between oxidative stress and necroptosis, a form of regulated necrosis, is controversial [72]. Some studies showed that ROS is the critical mediator of necroptosis [73], but other studies showed that ROS scavengers failed to prevent necroptosis in certain cell types [74]. In pyroptosis, the release of pro-inflammatory contents is initiated by the formation of inflammasome. ROS can activate inflammasome by sensitizing the NF-κB-pathways [75]. Oxidative stress is most closely associated with ferroptosis [76]. Ferroptosis is characterized by the increased accumulation of oxidative stress that leads to cell death. The increased oxidative stress induces the release of Fe(II) from iron compounds. The up-regulated availability of iron leads to the lipid peroxidation that culminates in ferroptosis [77].

## 4. Oxidative Stress and Cardiovascular Disease 

**Figure 2:**
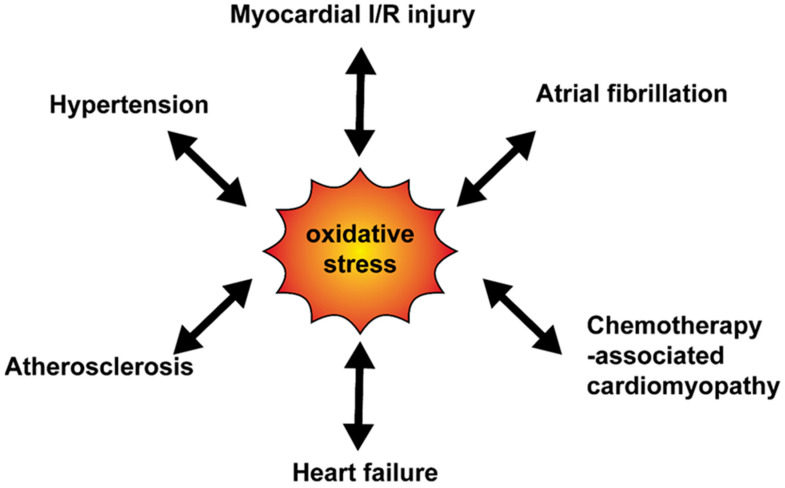
The relationship between oxidative stress and cardiovascular diseases. Various cardiovascular diseases enhance the oxidative production and at the same time, oxidative stress mediates progress of diseases. I/R, ischemia-reperfusion.

The relationship between oxidative stress and cardiovascular diseases is shown in Figure 2.

### 4.1. Oxidative Stress and Myocardial Ischemia-Reperfusion (I/R) Injury

Myocardial I/R injury is characterized by restoration of blood flow to the oxygen-deprived organs. The rapid re-establishment of blood flow leads to the oxygen burst and the ROS overproduction [78]. It is one of the most important pathogenic mechanism in acute coronary syndrome, myocardial infarction, surgical coronary bypass surgery, coronary revascularization intervention, circulatory shock, or organ transplantation [79]. Myocardial I/R injury accounts for approximately 25% of cell deaths during myocardium infarction [80]. The reperfusion injury can cause the “no-reflow” phenomenon, myocardial stunning, reperfusion arrhythmias, and reperfusion injury [81]. 

During reperfusion, the sources of ROS overproduction are mitochondria **[82]**, NOX family [83], Xo [84], and uncoupled NOS [85]. Complex I and III are the major sites for ROS overproduction in myocardial I/R injury. In addition, mPTP participates in the regulation of ROS overproduction in mitochondria. Braunersreuther et al. showed that the infarcted myocardium was reduced in the NOX1 and the NOX2 deficient mice compared with the wild-type mice, while NOX4 deficient mice had no obvious phenotype [86]. At the same time, redox signaling, including the hypoxia-inducible factor (HIF) pathway [87] and Nuclear factor E2-associated factor 2 (Nrf2) [88] pathway, is also activated to antagonize the ROS burst. HIF is an oxygen sensitive transcription factor which is also regulated by NOX-related ROS production. HIF-1α attenuates I/R injury through the regulation of inducible NOS, heme oyxgenase-1, cyclooxygenase-2, and antioxidant enzymes. Studies by Li et al. showed that HIF also directly targets mitochondria and have a protective role [89]. Nrf2 is a family of transcription factors. It is located in the cytosol under normal conditions. During the oxidative stress, it is translocated into the nucleus to regulate the expression of antioxidant and anti-inflammatory factors [90].

Increased ROS is associated with cardiomyocyte mitochondria damage, DNA damage, and protein degradation, which all lead to irreversible cell death [91]. Mitochondria damages are considered as the central process of oxidative stress-mediated myocardial I/R injury. Lochner et al. showed that mitochondrial depolarization resulted in mitophagy. However, the repressed mitophagy triggered the impairment of ATP production and Ca^2+^ overload [92]. Myocardial I/R injury is also associated with abnormal opening of mPTP, which could lead to apoptosis or necrosis. Cyclophilin D (CyPD) resides in the mitochondrial matrix working as a scaffold to control mPTP. Evidences show that S-nitrosylation modifications of CyPD is regulated by oxidative stress; CyPD knock-in mice show less I/R injury [93].

Endoplasmic reticulum stress is also regulated by oxidative stress and plays an important role in myocardial I/R injury [94]. Oxidative stress modifies amino acid residues to regulate protein activities and disturb intracellular Ca^2+^-homeostasis. In addition, inflammation is of great importance in myocardial I/R injury process. After myocardial I/R injury, cytokine cascades, such as tumor necrosis factor-α (TNF-α) and interleukin-1β (IL-1β), are activated. The activated cytokines will induce inflammation in these cells. Among them, neutrophils are the predominant early responder [95]. Neutrophils recruited to the infarct zone contain high levels of NOX2 and MPO, which facility the production of ROS. Furthermore, cytokine cascade has been shown to suppress cardiac contractility and decrease collagen synthesis [96].

A clinical study in patients undergoing the primary percutaneous coronary intervention showed plasma 8-iso-prostaglandin F2alpha, which is used as an indicator of oxidative stress, was increased after the procedure. However, there was no relationship between 8-iso-prostaglandin F2alpha and troponin T [97]. In patients undergoing coronary artery bypass surgery, thiobarbituric acid reactive substances (TBARS) was measured to indicate the oxidative stress status. The results showed that the oxidative stress was increased after surgery and the peak increase was seen 1 h after the reperfusion [98].

### 4.2. Oxidative Stress and Heart Failure (HF)

HF is characterized by the inadequate cardiac output to meet the bodily demands. Clinically, it is manifested by shortness of breath and/or chest tightness [99]. Studies showed that ROS are overproduced in all stages of HF [100]. Interestingly, the main source of ROS is different in HF patients with or without reduced ejection fraction. HF with reduced ejection fraction (HFrEF) is characterized by a reduced ejection fraction and is commonly due to coronary artery disease (CAD). In HFrEF, the injured cardiomyocytes produce ROS which leads to the maladaptive remodeling through programmed cell death and fibrosis [101]. In contrast, HF with preserved ejection fraction (HFpEF) is usually caused by hypertension, diabetes or genetics (e.g., hypertrophic cardiomyopathy). In HFpEF, the endothelial cells are the major sites of ROS overproduction [101,102]. The risk factors associated with HFpEF result in elevated plasma proinflammatory factors, such as IL-6, soluble ST2, and TNF-α. These factors act on the endothelial cells to induce ROS production, which upregulate the protein kinase G signaling in cardiomyocytes.

Mitochondria are major sites of ROS production in failing hearts while NOX and Xo activities are also increased [103,104]. The overproduction of ROS in HF patients causes mitochondria damage, which gives feedback to produce more ROS [105]. At the same time, ROS accelerates myocardial remodeling by activating variety of hypertrophic signaling kinases and transcription factors, such as tyrosine kinase Src, GTP-binding protein Ras, and mitogen-activated protein kinases (MAPKs) [106,107]. In addition, matrix metalloproteinases, important factors for myocardial structure, are also activated by ROS [108].

It is worth noting that the chemotherapy associated cardiomyopathy is a special kind of HF. It is relatively common and serious complication of chemotherapy [109]. Many classes of chemotherapeutic agents that are widely used in the clinics are identified to have cardiac toxicity, such as anthracyclines and alkylating agents [110]. These agents cause the increase in ROS generation and enhance the oxidative stress in the cell, which then leads to cardiomyocyte death [111]. Doxorubicin (DOX), a widely used anthracycline chemotherapeutic drug, directly modifies the mitochondrial DNA and disturbs the mitochondrial function, the protein expression, and the lipid peroxidation [111]. DOX combines with free iron to form iron-Dox complex, then reacts with oxygen and facilitates ROS production [112].

Clinical study by Tedgui showed that pericardial levels of 8-iso-prostaglandin F2alpha was associated with severity of HF [113]. The study by Chopra et al. investigated plasma lipid peroxides (MDA) in congestive HF patients and found an inverse correlation between MDA and left ventricular ejection fraction [114].

### 4.3. Oxidative Stress and Atherosclerosis

Atherosclerosis is the underlying pathology of ischemia heart diseases, stroke and peripheral artery diseases. Oxidative stress is essential for the pathological progress of atherosclerosis. Development of atherosclerotic plaques will decrease the oxygen supply which is the basis of many kinds of cardiovascular diseases [115]. Atherosclerosis is initiated by the injury of endothelial cells, followed by the infiltration and accumulation of oxidized low-density lipoprotein (ox-LDL) cholesterol to the subendothelial space. At the same time, leukocytes migrate to the subendothelial space. Monocytes-originated macrophages engulf ox-LDL to form foam cells [116]. NOX, Xo, mitochondrial enzymes are mainly responsible for the production of ROS in atherosclerosis [117]. NOX1 [118] and NOX4 [119] are detected in vascular smooth muscle cells (VSMCs). In comparison, NOX2 [120], NOX4 [121], and Xo [122] are found in endothelial cells.

Oxidative stress regulates the pathophysiology of atherosclerosis at all stages. First, oxidative stress causes endothelial dysfunction by altering endothelial signal transduction and redox-regulated transcription factors, which increase vascular endothelial permeability and catalyze leukocyte adhesion. This is considered as the initiation of plaque formation. Then, plasma LDL is recruited to the arterial wall where it is modified by oxidative stress to form ox-LDL. Ox-LDL can be taken up by macrophage to form foam cells. In addition, oxidative stress alters the expression of adhesion molecules, such as a vascular cell adhesion molecule-1, to regulate adhesion of monocytes. At the same time, increased ROS stimulate the development of the plaque by enhancing VSMCs migration and collagen deposition. Finally, oxidative stress exacerbates the stability of the plaque by releasing matrix metalloproteinase to degrade the fibrous wall [123,124].

Channon et al. showed the association between endothelial dysfunction and increased vascular superoxide production in human atherosclerosis [125]. Other study demonstrating the increase in erythrocyte TBARS with the severity of obstruction of the artery supports the potential causal relationship between oxidative stress and atherosclerosis [126].

### 4.4. Oxidative Stress and Atrial Fibrillation (AF)

AF is the most common arrhythmia in clinical practice with symptoms of irregular and rapid heart rate [127]. In rats, decreased plasma antioxidant capacity was associated with increased risk of AF [128]. Quyyumi et al. showed that there were elevated cystine level, cystine/glutathione ratio, and redox potential of glutathione (all indictive of increased oxidative stress) in AF patients [129]. The sources of ROS include NOX2/4 enzymes, which is upregulated in fibrillating area, and Xo [130].

There may be multiple mechanisms for how ROS causes AF. First, the increase in ROS regulates ionic leaks in cardiomyocytes. It increases Na^+^ current [131], L-type Ca^2+^ current [132], and Ca^2+^ leak from the sarcoplasmic reticulum (SR) [133]. All these result in prolonged action potential duration and reduced conduction velocity [134]. Furthermore, oxidative stress promotes myocardial fibrosis by facilitating the deposition of collagen [135]. The myocardial fibrosis interferes with the electrical coupling of myocytes [136]. Finally, oxidative stress may cause AF by regulating iron current associated proteins, DNA and post-translational modifications. Angiotensin II and hypoxia result in Na^+^ current abnormality by regulating Na^+^ voltage-gated channel alpha subunit 5 (SCN5A) splicing mRNA. Ca^2+^/CaM-dependent kinase II (CaMKII) is oxidized by ROS and has potential to regulate Na^+^ current by ryanodine receptor, an important Ca^2+^ control protein located in the SR [137,138].

### 4.5. Oxidative Stress and Hypertension

Hypertension is regarded as the major risk factor for cardiovascular diseases. It is a pathologic blood pressure increase resulting from the abnormal vasorelaxation factor levels. Furthermore, laboratory studies showed that oxidative stress levels in hypertension models differ from control group [139,140]. Similar to atherosclerosis, NOX families are regarded as a major source of ROS while Xo, NOS, mitochondria also have important roles in ROS increase [62,124]. Oxidative stress regulates hypertension by targeting endothelial cells. Vascular tonicity is regulated by the balance of endothelium-derived relaxing (EDRFs) factors and endothelium-derived hyperpolarizing factors (EDHFs). ROS is known as a member of EDHFs, whereas nitric oxide (NO) is a member of EDRFs [141]. ROS is able to decrease the bioavailability of NO and increase the amount of endogenous endothelial NOS antagonist, such as asymmetric dimethylarginine (ADMA). Additionally, endothelial function is regulated by cell phosphorylation pathways, such as tyrosine kinases, phosphoinositol-3-kinase/Akt kinase (PI3K/Akt) and the MAPKs, and the gene expression factors, such as p53 and activated protein-1 (AP-1). All these pathways and gene expression factors can be initiated and controlled by oxidative stress [123,142].

Chayama et al. measured urinary excretion of 8-hydroxy-2′-deoxyguanosine and serum MDA-modified LDL as the indicator of oxidative stress in patients with renovascular hypertension. They showed that there was an increase in oxidative stress indicators in these hypertensive patient compared to the control group without hypertension [143]. Another study by Beevers also showed that the lipid hydroperoxides were upregulated in hypertension patients [144].

## 5. Therapies for Oxidative Stress-Associated Cardiovascular Diseases

### 5.1. Antioxidant Molecules

#### 5.1.1. Nutritional Supplements

Accumulating evidences demonstrate that many nutritional supplements have antioxidant properties [145]. Vitamin A is a series of unsaturated nutritional organic compounds and can react with oxidative species [146]. Vitamin A has shown to modify the effect of apolipoproteins on the risk of myocardial infarction [147]. It is important to note that carotenoids are the precursors of vitamin A, and astaxanthin, one of the most notable carotenoids, is regarded as a ROS scavenger. Preclinical studies of astaxanthin demonstrated the protective effect in I/R injury and thrombotic diseases in animal models [148]. Moreover, astaxanthin has been shown to decrease blood pressure in spontaneously hypertensive rats [149]. In a randomized double-blinded clinical trial, the intake of astaxanthin reduced the serum lipid peroxidation biomarker levels while increasing the SOD level [150].

Vitamin C can detoxify exogenous and endogenous ROS as well as the modified proteins and lipids that were modified by the ROS. Studies have shown that vitamin C could control endothelial cell proliferation and apoptosis and smooth muscle-mediated vasodilation, which are both important in the pathogenesis of cardiovascular diseases [151]. Vitamin E inhibits superoxide production by impairing assembly of NOX enzymes. Experimental studies have shown that vitamin E can reduced the risk of coronary heart disease and decreased the cardiovascular complications. A meta-analysis of 400,000 patients reported that the rate of coronary heart disease was decreased by vitamin E and vitamin C intake [152].

Omega-3 represents an attractive strategy to reduce the susceptibility to oxidative stress injury in myocardial cells by modulating redox pathways. In a rat model of myocardial infarction, rats supplemented with omega-3 showed lower infarct size [153]. Flavanols, such as quercetin, are reported to decrease oxidative stress markers and improve cardiac function in both animal models and patients with cardiovascular diseases [154]. A clinical trial with 805 elderly people reported that the mortality from coronary heart disease was decreased by high flavonoid intake [152].

On the other hand, a meta-analysis (includes 66 randomized trials) examining the effect of nutritional compounds to treat cancer or cardiovascular diseases showed mixed results. Out of 66 trials examined, 24 showed a positive outcome, 39 showed a negative outcome, and 3 showed a neutral outcome [7]. The positive outcome was mostly observed in participants who were regarded as malnutrition. These findings suggest that the status of nutrition of patients will affect the efficacy of antioxidant supplementation. It is effective for those patients who lack certain nutrients that contribute to the antioxidant defense network. Whether the additional intake will have beneficial effects in those patients who are not deficient in these nutrients are unclear at this time.

#### 5.1.2. Novel Experimental Antioxidant-Based Therapies

There are novel antioxidants that can be divided into following three categories: the activators of endogenous antioxidant defense systems, the inhibitors of oxidative stress formation, and the compounds that allow functional repair of ROS-induced damage. NRF2 activators is an example of the activators of endogenous antioxidant defense systems. NRF2 is a basic transcription factor that recognizes the enhancer called Antioxidant Response Element. The individuals with decreased NRF2 expression and activity are more likely to develop atherosclerosis or hypertension [155]. In Nrf2 KO mice, the cardiac structure and function were impaired, and these mice were more susceptible to develop HF [155]. The most well-studied drug that targets NRF2 is dimethyl fumarate (DMF). It is already in clinical use for the treatment of multiple sclerosis. In vivo experiments showed that DMF reduced infarction size after I/R injury [156]. In vitro experiments have also indicated its protective role in cardiomyocytes after I/R injury [157]. Additionally, in the apolipoprotein E (apo-E)-deficient mouse model with streptozotocin-induced hyperglycemia, DMF reduced the development of atherosclerosis [158].

The second category of compounds are the inhibitors of oxidative stress formation include drugs targeting Xo, NOX, and MPO. Xo inhibitor, allopurinol, is a promising therapeutic agent. Although large-scale prospective studies evaluating allopurinol in cardiovascular diseases are still lacking, small clinical studies have indicated a beneficial effect of allopurinol in hypertension, I/R injury and HF by limiting the oxidative stress in endothelial cells [159]. A meta-analysis including 10 clinical studies evaluating the effect of allopurinol on blood pressure also showed a modest reduction of blood pressure [160]. In addition, the studies evaluating allopurinol in patients undergoing coronary artery bypass grafting or primary percutaneous coronary intervention showed a reduced in-hospital mortality and cardiac complications [159]. As for NOX inhibitor, GKT137831 is the first NOX inhibitor in clinical development [161]. The treatment using GKT137831 resulted in a profound anti-atherosclerotic effect in apo-E KO mice [161]. GKT137831 also rescued cardiac function after I/R injury in mice [162]. In mice model of atherosclerosis, MPO inhibitors were able to alter the atherosclerotic lesion composition and cardiac remodeling [163].

The last group is the compounds that allow functional repair of ROS-induced damage, which mainly focuses on nitric oxide-cyclic guanosine monophosphate (NO-cGMP) signaling. ROS can interfere with NO-cGMP signaling through uncoupling NOS, chemically scavenging NO, or oxidatively damaging the NO receptor (soluble guanylyl cyclase, sGC). The drugs in this group include HNO donors, such as CXL-1427, which is the second generation of HNO donor compounds. A Phase 2a dose-escalation study showed a favorable safety profile and hemodynamic effects in hospitalized patients with HFrEF [164]. L-citrulline and L-arginine are also included in this group since they are able to recouple the NOS [165]. A meta-analysis of 11 randomized, double-blinded, placebo-controlled trials showed that oral administration of L-arginine was able to lower the systolic blood pressure by 5.39 mmHg and the diastolic blood pressure by 2.66 mmHg. Meta-analysis of 15 randomized controlled trials with 424 participants showed that L-citrulline administration resulted in 7.54 mmHg reduction in the systolic blood pressure and 3.77 mmHg reduction in the diastolic blood pressure [166].

#### 5.1.3. Antioxidant Role of Clinical Drugs

**Figure 3:**
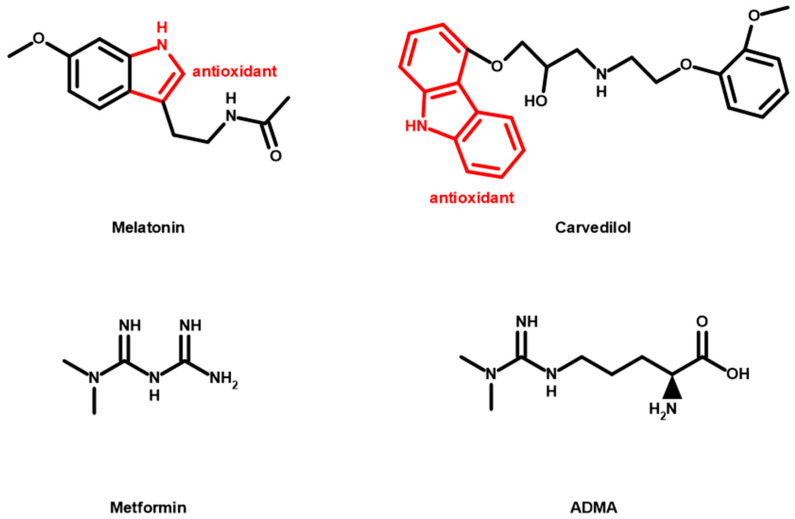
Molecular structures of melatonin, carvedilol, metformin, and asymmetric dimethylarginine (ADMA).

There are drugs that are currently being used in a clinical setting that are known to have antioxidants effects, such as melatonin, proprotein convertase subtilisin/kexin type 9 (PCSK9) inhibitor, carvedilol, and metformin. Melatonin is a potent free radical scavenger because of its antioxidant properties [167] and has shown to have a protective role in myocardial I/R injury, HF, and atherosclerosis via targeting oxidative stress in animal studies [168]. PCSK9 inhibition decreases the ROS production in endothelial cells and smooth muscle cells by inhibiting lectin-like oxidized low-density lipoprotein receptor-1 (LOX-1) expression in mice atherosclerosis models [169]. Carvedilol is a combined β1-, β2-, and α1-adrenergic blocking agent that has antioxidant properties [170]. It is important in the treatment of HF [171]. Metformin reduces ROS production by inhibiting NOX pathway and increasing antioxidant genes. Furthermore, because metformin is a structural analog of ADMA (Figure 3), it can help regulate the balance between NO and ADMA [172]. Both the animal studies and the clinicals trials showed protective role in CAD and HF [173]. Molecular structures of some of these clinical drugs are shown in Figure 3.

### 5.2. miRNAs

miRNAs are series of small noncoding RNAs ascribed to regulate gene expression by targeting messenger RNAs. Accumulating evidence shows that miRNAs are involved in oxidative stress response and are critical in regulating oxidative stress. miRNA-210 is a well-known hypoxia-induced RNA which is significantly upregulated during hypoxia [174]. miRNA-210 is considered as the most significant anoxic-related miRNA in the body; it improves cardiac function by inhibiting apoptosis and promoting angiogenesis [175]. The expression of miRNA-210 is mainly induced by HIF-1α [176]. In the HIF-1α knockout mice, the miRNA-210 level was decreased compared to the wild-type mice [177]. In the infarcted myocardial tissues of patients who died from acute myocardial infarction, miRNA-210 level was increased compared with the heart tissues from the control group [178]. In animal model of myocardial infarction, the cardiac function was improved by a direct myocardial injection of miRNA-210 [179]. In addition, circulating miRNA-210 level showed significant association with cardiovascular-related mortality in patients presenting with acute coronary syndrome [180].

miRNA-1 is the most abundant miRNA expressed in cardiac muscles and plays a key role in differentiation and proliferation of muscle cells. H_2_O_2_ was found to increase miRNA-1 in rat cardiomyocytes [181]. Overexpression of miRNA-1 resulted in increased ROS and decreased production of SOD [181]. In a rat model of myocardial infarction, the amount of miRNA-1 was positively associated with infarct size [182]. In addition, administration of miRNA-1 after myocardial infarction improved cardiac function in mice [183]. In addition, serum levels of miRNA-1 in patients with acute coronary syndrome correlated with the circulating troponin T [184]. Other miRNAs, such as miRNA-132, miRNA-21, and miRNA-17, are also shown to be upregulated during hypoxia [185,186,187].

Additionally, many miRNAs are important in the regulation of atherosclerotic plaque formation [188]. The cross-sectional observational study with 100 subjects showed an increase in miRNA-133 level in the patients with CAD compared with the control group [189], and the miRNA-133 level was increased in symptomatic plaques [190]. In addition, inhibition of miRNA-133 can target NOS to prevent endothelial dysfunction [191]. miRNA-92a can regulate the expression of endothelial NOS to affect endothelial cells [192]. miRNA-92a has been shown to reduce plaque inflammation and increased the plaque stability by promoting endothelial cell proliferation and angiogenesis [193]. However, because of low stability and bioavailability, a lot of work are still needed to make miRNAs a feasible therapeutic option. For future clinical application, multiple strategies based on inducing or repressing miRNA expression, such as the use of miRNA antagonists or mimics, are also being examined.

### 5.3. Nanoparticles

Nanomedicine is a field of science that uses nanomaterials for the diagnosis and treatment of human disease [194]. Nanoparticles are attractive because of their size and their properties that allow easy modification [8]. Our group developed novel H_2_O_2_-responsive nanoparticles that could specifically target the site of I/R injury, where H_2_O_2_ is the dominant oxidative species [195]. These nanoparticles are generated from co-polyoxalate and vanillyl alcohol (VA), an antioxidant extracted from natural herb. They contain H_2_O_2_-responsive peroxalate ester linkage that rapidly degrade at the site of high H_2_O_2_ concentration, and releases VA that exerts anti-inflammatory and anti-apoptotic activities. In various animal models of acute I/R injury, these nanoparticles demonstrated potent anti-inflammatory and anti-apoptotic activities resulting in reduced organ damages [195,196]. These nanoparticles also effectively reduced DOX-induced cardio and hepato-toxicities in vivo, which resulted in significant increase in survival outcome [197].

Other studies used nanoparticles to decrease oxidative stress by targeting oxidative stress production or clearance system. Somasuntharam et al. used nanoparticles coated with NOX2 small interfering RNA (siRNA) and injected directly into the myocardium in mice after an experimental myocardium infarction. They observed improved cardiac function 3 days after the surgery [198]. Since SOD has a protective role, nanoparticles designed to carry SOD1 were also injected at the ischemic zone in a rat I/R injury model. This therapy resulted in decreased myocyte apoptosis and improved cardiac function [199]. Therapy with the nanoparticles designed to carry N-acetylcysteine also showed effective attenuation of cardiac fibrosis in a rat I/R model [200]. In addition, studies using nanoparticle-based delivery of selenium, a metal oxide that has ROS-quenching properties, showed improved biological effect in ischemic cardiomyocytes [201]. However, although promising, the clinical application of nanomedicine in cardiovascular diseases is still in infancy. All of the therapies targeting oxidative stress are summarized in Table 2.

### 5.4. Limitation

Although oxidative stress plays an important role in various cardiovascular diseases, application of antioxidant therapy so far has been limited in the clinical settings. First, although suppression of oxidative stress using antioxidants has been shown to beneficial in many animal models, the beneficial effects of these antioxidant therapies in human clinical studies have been controversial. One of the reasons may be due to non-specific suppression of ROS, which may not be desirable or effective because it could disrupt important ROS-mediated cellular signaling. Therefore, targeted suppression at the site of ROS overproduction, (e.g., in heart for myocardial infarction), such as using targeted nanoparticles, may offer more effective antioxidant therapy [195].

In addition, the dynamic character of the disease makes it important to choose an appropriate time to use the antioxidants. At different stages of diseases, oxidative stress may have different roles. Thus, it is crucial to give the antioxidative treatments at the appropriate time. Finally, for novel compounds, most of the studies are based on the laboratory experiments. More large-scale clinical trials with various cardiovascular patients are needed at this time [207,208].

### 5.5. Novelty

This article focuses on the relationship between oxidative stress and cardiovascular diseases, and discusses the current status of different antioxidant drugs that are being used or being studied for cardiovascular diseases. Furthermore, we included novel strategies that have potential to be used in future therapies.

## 6. Conclusions

Oxidative stress plays an important role in the development and the evolution of cardiovascular diseases. Various therapeutic strategies targeting oxidative stress have been developed. Although animal studies have shown beneficial effects of antioxidant therapy in various cardiovascular diseases, the clinical outcomes vary in human trials. The deeper understanding of oxidative stress in the cardiovascular diseases and development of better antioxidants therapies are needed to have more effective treatments of cardiovascular diseases.

## Figures and Tables

**Table 1:** Description of antioxidant defense enzymes.

Compound: Isoforms	Effects	Research Type: Subjects	Main Findings	Ref
SOD: Cu/ZnSOD, MnSOD, ECSOD	Accelerates the reaction of superoxide anion to form H_2_O_2_ and oxide.	Preclinical: mice	Cu/ZnSOD-deficiency resulted in altered responsiveness in both large arteries and microvessels.	[22,28]
		Preclinical: Rabbits	Gene transfer of ECSOD reduced infarct size.	[29]
		Clinical: HTN patients	Serum levels of SOD were associated with alterations in vascular structure and function.	[30]
Catalase	Lower H_2_O_2_ concentration: accelerate the reaction of H_2_O_2_ with hydrogen donors to produce water	Preclinical: mice	Overexpression of catalase prevented HTN.	[25,31]
GPx: GPx 1–8	Catalyze H_2_O_2_ or organic hydroperoxides to water or corresponding alcohols.	Preclinical: mice	GPX knockout mice were more susceptible to I/R injury.	[27,32]
		Preclinical: mice	Deficiency of GPX accelerated atherosclerotic lesion progression.	[33]
		Clinical: CAD patients	GPX-1 Pro198Leu polymorphism was higher in patients with CAD.	[34]
GR	Clear the oxidized dimer form of glutathione to reduced glutathione.	Clinical: CAD patients	Highest GR activity was associated with myocardial infarction.	[35,36]
Prx: 2-Cys, atypical 2-Cys, and 1-Cys Prx	Catalyze H_2_O_2_ or organic hydroperoxides to water or corresponding alcohols.	Preclinical: mice	Overexpression of Prx-3 inhibited left ventricular remodeling and HF after myocardial infarction.	[37,38]
		Preclinical: mice	Prx1 protected against excessive endothelial activation and atherosclerosis.	[39]
		Clinical: HF patients	Plasma PRX was higher in HF patients.	[40]
MSR: (1) MSRA and MSRB (2) fRMSR and MSRP (3) MPT/WPT OR enzymes	Reduce methionine sulfoxide residues in oxidatively damaged proteins to methionine residues.	Preclinical: mice	Hepatic overexpression of MSRA reduced dyslipidemia and atherosclerosis.	[41,42]
		Preclinical: mice	Cytosolic MsrA protected the heart from I/R injury.	[43]
		Clinical: CAD patients	MSR was associated with etiology of CAD.	[44]
Trx	Transfer electrons to Prxs, MSRs, other redox-sensitive transcription factors.	Preclinical: mice	Overexpression of Trx reversed aged-related HTN.	[45,46]
		Preclinical: mice	Inhibition of endogenous cardiac Trx1 stimulated hypertrophy.	[47]
		Clinical: General population	Trx80 increased in aged people.	[48]
Grx: Grx 1–5	Catalyze the reduction of protein disulfides or mixed disulfides, and maintain the intracellular redox status.	Preclinical: mice	Grx-1 diminished ventricular remodeling in chronic myocardial infarction	[49,50]

SOD, superoxide dismutase; Cu/ZnSOD, copper-zinc SOD; MnSOD, manganese SOD; ECSOD, extracellular SOD; HTN, hypertension; GPx, glutathione peroxidase; I/R, ischemia-reperfusion; CAD, coronary artery disease; GR, glutathione reductase; Prx, peroxiredoxin; HF, heart failure; MSR, methionine sulfoxide reductases; MPT, molybdopterin; WPT, tungstopterin; OR, oxidoreductase; Trx, thioredoxin; Grx, glutaredoxin.

**Table 2:** Description of antioxidant therapies.

Compound	Research Type: Subjects	Main Finding	Ref
**Nutritional Supplements**			
Vitamin A	Clinical: Stable angina patients	Modified the effect of apolipoproteins on the risk of MI	[147]
Astaxanthin	Preclinical: Dogs	Astaxanthin protected from MI	[202]
	Preclinical: Rats	Astaxanthin reduced HTN in spontaneously hypertensive rats	[149]
	Clinical: Obese adults	The supplemental of astaxanthin decreased oxidative stress	[150]
Vitamin C	Clinical: CHF patients	Vitamin C inhibited endothelial cells apoptosis in CHF patients	[203]
Vitamin E	Clinical: General population	The intake of vitamin E reduced risk of coronary heart disease	[204]
Vitamin C+ vitamin E	Meta-analysis: general population	Vitamin E and vitamin c combination inhibited the rate of coronary heart disease.	[152]
Omega-3	Preclinical: Rats	The supplement of omega-3 was associated with lower infarct size	[153]
Flavanols	Preclinical: Rats	Flavanols reduced the MI size and fat peroxidation	[205]
	Clinical: HTN patients	Flavanols reduced the mean blood pressure in HTN patients	[154]
	Clinical: CVD patients	Flavonoid reduced coronary heart disease mortality.	[152]
Multiple supplements	Meta-analysis: Cancer or CVD patients	Nutritional supplements showed protective in malnutrition patients.	[7]
**Novel Experimental Antioxidant-Based Therapies**			
NRF2 activators	Preclinical: Knockout mice	In Nrf2 knockout mice, cardiac structure and function were impaired.	[155]
DMF	Preclinical: Rats	DMF reduced MI size.	[156]
	Preclinical: Mice	DMF reduced development of atherosclerosis in diabetes mice model	[158]
Allopurinol	Meta-analysis: HTN patients	Allopurinol showed a modest reduction of blood pressure	[160]
	Clinical: CABG patients	Allopurinol showed reduced in-hospital mortality and cardiac complications	[159]
GKT137831	Preclinical: Mice	GKT137831 resulted in anti-atherosclerotic effect	[161]
	Preclinical: Mice	GKT137831 rescued cardiac function after I/R injury	[162]
MPO inhibitors	Preclinical: Mice	MPO inhibitors showed utility to stabilize atherosclerotic lesion	[163]
CXL-1427	Clinical: HF patients	CXL-1427 showed a favorable safety and hemodynamic effect	[164]
L-citrulline	Meta-analysis: HTN patients	Administration of L-citrulline lowered blood pressure	[166]
L-arginine	Meta-analysis: HTN patients	Administration of L-arginine lowered blood pressure	[166]
**Clinical Drugs**			
Melatonin	Clinical: CAD patients	Melatonin decreased CK-MB in patients undergoing primary percutaneous procedure	[168]
PCSK9 inhibitor	Preclinical: Mice	PCSK9 inhibition decreased ROS	[169]
Carvedilol	Clinical: General population	Carvedilol significantly inhibited ROS generation	[171]
Metformin	Preclinical: Rats	Metformin showed antihypertensive effect in spontaneously hypertensive rats by restoring ADMA-NO balance	[172]
**miRNAs**			
miRNA-210	Preclinical: Knockout mice	miRNA-210 was decreased by HIF-1α knockout	[177]
	Preclinical: Mice	The intramyocardial injection of miRNA-210 improved cardiac function after MI	[179]
	Clinical: Acute MI patients	miRNA-210 level was increased patients with MI	[178]
	Clinical: ACS patients	miRNA-210 level was associated with cardiovascular-related mortality	[180]
miRNA-1	Preclinical: Transgenic mice	miRNA increased ROS and decreased production of SOD	[181]
	Preclinical: Rat	miRNA-1 was associated with MI size	[182]
	Preclinical: Mice	The post-infarction transplantation of miRNA-1 improved cardiac function	[183]
	Clinical: MI patients	Serum levels of miRNA-1 in patients with acute coronary syndrome correlated with the circulating troponin T	[184]
miRNA-133	Preclinical: Mice	Inhibition of miRNA-133 prevented endothelial dysfunction	[191]
	Clinical: CAD patients	miRNA-133 was higher in CAD patients	[189]
	Clinical: Patients undergoing carotid endarterectomy	miRNA-133 level was increased in symptomatic plaques	[206]
**Nanoparticles**			
H_2_O_2_-responsive nanoparticles	Preclinical: Mice	H_2_O_2_-responsive nanoparticles showed anti-apoptotic role in hind-limb I/R and liver I/R models	[195]
	Preclinical: Mice	H_2_O_2_-responsive nanoparticles showed protective role in myocardial I/R injury	[196]
	Preclinical: Mice	H_2_O_2_-responsive nanoparticles showed protective role in doxorubicin-induced cardiomyopathy	[197]
Nanoparticles/NOX2 siRNA	Preclinical: Mice	Nanoparticles coated with NOX2 siRNA improved cardiac function 3 days after surgery	[198]
Nanoparticles/SOD1	Preclinical: Rat	Nanoparticles carrying SOD1 decreased myocyte apoptosis and improved cardiac function	[199]
Nanoparticles/N-acetylcysteine	Preclinical: Rat	Nanoparticles carrying N-acetylcysteine attenuated cardiac fibrosis after I/R injury	[200]

MI, myocardial infarction; HTN, hypertension; CHF, chronic heart failure; CVD. Cardiovascular disease; NRF2, nuclear factor E2-associated factor 2; DMF, dimethyl fumarate; CABG, coronary artery bypass grafting; I/R, ischemia-reperfusion; MPO, myeloperoxidase; CAD, coronary artery disease; PCSK9, proprotein convertase subtilisin/kexin type 9; ROS, reactive oxygen species; ADMA, asymmetric dimethylarginine; HIF, hypoxia-inducible factor; SOD, superoxide dismutase; NOX2, nicotinamide adenine dinucleotide phosphate (NADPH) oxidase 2.
